# Presenilin‐2 knock‐In mice show severe depressive behavior via DVL3 downregulation

**DOI:** 10.1111/cns.14370

**Published:** 2023-07-27

**Authors:** Seung Sik Yoo, Dong Won Lee, Hyeon Joo Ham, In Jun Yeo, Ju Young Chang, Jaesuk Yun, Dong Ju Son, Sang‐Bae Han, Jin Tae Hong

**Affiliations:** ^1^ College of Pharmacy and Medical Research Center Chungbuk National University Cheongju South Korea; ^2^ Ministry of Food and Drug Safety (MFDS) Cheongju South Korea; ^3^ Korea Health Industry Development Institute Cheongju South Korea

**Keywords:** Alzheimer's disease, depression, DVL3, GSK3β, PS2, tau

## Abstract

**Introduction:**

Alzheimer's disease (AD) is the most common form of dementia. Depression is one of the most critical psychiatric complications of AD, and 20%–30% of patients with AD experience symptoms of depression. Phospho‐glycogen synthase kinase‐3 beta (GSK3β) is known to be associated with AD and depression. Furthermore, the role of disheveled (DVL) is known to regulate GSK3β. Moreover, presenilin‐2 (PS2) and DVL have cross‐talk with each other. Also, it is widely hypothesized that stress leads to hypersecretion of cortisol and is thus associated with depression. Dickkopf WNT signaling pathway inhibitor‐1 (DKK‐1) is a crucial factor regulating depression and both amyloid beta (Aβ) and phosphorylation of tau are widely known as a biomarker of AD.

**Methods:**

To investigate the relationship between AD and depression, and possible pathways connecting the two diseases, we examined memory function and depression‐related behavior test results in PS2 knock‐in AD mice (PS2 MT). Next, we confirmed that there are relationships between DVL, depression, and cognitive disease through the comparative toxicogenomics database (https://ctdbase.org) and STRING (https://string‐db.org) database.

**Results:**

PS2 knock‐in mice showed much more severe memory impairment and depression than PS2 wild‐type mice (PS2 WT). In AD‐related behavioral experiments, PS2 MT mice showed more memory dysfunction compared with PS2 WT group mice. Moreover, Aβ and phosphorylation of tau showed higher expression in PS2 MT mice than in PS2 WT mice. Depression‐related behavioral tests showed that PS2 MT mice exhibited more depressive behaviors than PS2 WT mice. Furthermore, both higher cortisol levels and higher expression of DKK‐1 were found in PS2 MT mice relative to PS2 WT mice. The results indicated that there is a relationship between DVL and the release of AD‐related mediators and expression of the depression‐related glucocorticoid receptor and DKK‐1. In the PS2 knock‐in group, DVL was significantly decreased compared with the PS2 WT group.

**Conclusion:**

Depression increases the risk of developing AD and other forms of dementia. Recent evidence indicates that depression symptoms could trigger changes in memory and thinking over time. However, it is recognized that there are no drugs to facilitate a full recovery for both AD and depression. However, our results suggest that AD and depression could be associated, and DVL could be a significant target for the association between AD and depression.

## Graphical abstract

PSEN2 knock‐in mice show less phosphorylation level of Ser9 glycogen synthase kinase‐3 beta and decreased expression level of disheveled (DVL3) compared with the PSEN2 WT group. The results indicate that DVL3 could be a significant key regulator of both Alzheimer's disease and depression.

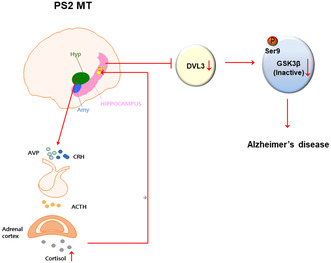

## 1 INTRODUCTION

Alzheimer's disease (AD) is a chronic neurodegenerative disease which is associated with memory deficits and cognitive decline, although less common clinical presentations are increasingly recognized.^1^ An estimated 6.2 million Americans aged 65 and older are living with Alzheimer's dementia today. This number could grow to 13.8 million by 2060 barring the development of medical breakthroughs to prevent, slow, or cure AD.^2^ Mutations in the amyloid protein precursor (APP), presenilin‐1 (PS1), and presenilin‐2 (PS2) genes are known as causes of AD.^3^ PS1 and PS2 genes are involved in amyloid beta (Aβ) synthesis and mutation of PS1 or PS leads to excessive production of Aβ.^4^ One of the mutations in the PS2 gene (N141I) has been described in early onset of AD.^5^ It was also found that PS2 (N141I) mutation increased Aβ generation, oxidative stress, and β‐secretase activity.^6^, ^7^, ^8^

Major depressive disorder (MDD) is a serious mental disorder characterized by a persistently depressed mood that severely affects a person's physical and mental health.^9^ It is also moderately heritable, so the age of relapse and early onset are associated with the greatest familial risk.^10^ It is widely known that chronic stress leads to increased secretion of cortisol and thus results in depression.^11^ There is research showing that cortisol infusion raises mood significantly in major depression.^12^ Moreover, cortisol levels and activated glucocorticoid receptor (GR) expression levels in the hippocampus of mice were increased by cold exposure stress.^13^ Depressed patients had significantly higher levels of GR phosphorylated at serine 211 (pGR‐Ser211) compared to healthy individuals.^14^ Dickkopf‐1 (DKK‐1) is an inhibitor of the canonical Wnt pathway that is involved in the chronic stress characteristic of MDD.^15^ Corticosterone also induces DKK‐1 expression via the activation of GRs, and damages neurons resulting in major depression and other stress‐related disorders in mouse models.^16^

Depression is now considered a relevant factor for the development of AD.^17^ It has been suggested that the increased incidence of depression in patients with AD points to a common neurobiological basis for both diseases.^18^ A large number of geriatric patients with MDD and a very high proportion of patients with AD present with cognitive deficits.^19^ Likewise, Geriatric Depression Scale scores are consistently a significant predictor of Dementia Rating Scale and Logical Memory scores.^20^ These data indicate that depression development could be associated with AD development.

Glycogen synthase kinase‐3 beta (GSK3β) plays an important role in AD.^21^ A great deal of research indicates that GSK3β is related to the hyperphosphorylation of tau, increased production of Aβ, and memory impairment.^22^, ^23^, ^24^ Like AD, GSK3β is associated with depression. Research shows that GSK3β‐mediated phosphorylation of tau plays a critical physiological role in long‐term depression (LTD).^25^ Other research shows that THY‐Tau22 mice can be rescued from LTD by inhibiting GSK3β.^26^ It has also been found that neurofibrillary tangles (NFTs) are more pronounced in the brains of AD patients with comorbid depression than in AD patients without depression.^27^ Thus, GSK3β is critical to both AD and depression development.

Disheveled (DVL) is a key protein closely related to GSK3.^28^, ^29^ DVL recruits the Axin/GSK3 complex to the plasma membrane and stimulates phosphorylation of GSK3.^30^ Overexpression of mouse DVL1 or DVL2 has been shown to inhibit GSK3β‐mediated phosphorylation of tau.^31^ Other research has indicated that APP‐PS1 AD mouse brains have lower levels of DVL3 and increased levels of activated GSK3β than WT mouse brains.^32^ Moreover, treatment with lithium, a GSK3β inhibitor, showed recovery levels of DVL3 accompanied by inhibition of GSK3β activity in APP‐PS1 AD‐related mouse brains.^33^ Other research indicates that DVL‐GSK3β signaling was activated in depression‐like behaviors.^34^ Overexpression of a dominant‐negative mutant of DVL in the nucleus accumbens, or the administration of a pharmacological inhibitor of DVL into mice brain region, blocks DVL function and renders mice more susceptible to social defeat stress and depression‐like behaviors.^35^, ^36^, ^37^ This research indicates that DVL‐GSK3β downregulation could be a significant factor mediating the link between AD and depression. However, whether PS2 knock‐in AD mice show depression‐like behavior and whether DVL is associated with PS2 mutation have not been studied. We therefore investigated the relationship between AD and depression in PS2 knock‐in mouse and the possible involvement of DVL signaling in the comorbidity of the two diseases.

## 2 METHODS

### 2.1 PS2 mutant transgenic mice

Twelve‐ to eighteen‐month‐old transgenic mice expressing wild‐type PS2 (PS2wt‐Tg) and mutant PS2 (PS2mt‐Tg) showed characteristics of AD (such as deposition of Aβ and memory loss) as described elsewhere.^7^, ^8^ Equal numbers of male and female mice including mutant PS2, first generated on a hybrid C57BL/6J × DBA2 background, and at least nine generations of the C57BL/6J strain from Korea Food and Drug Administration (KFDA) were used. The genotype was confirmed by PCR after tail biopsies, and the insertion of both gene‐coding sequences replaced the coding sequence of the human PS2 gene, allowing neuron‐specific enolase expression of the transgene. It was also demonstrated that the PS2 levels are similar in wild‐type and mutant PS2 mice as described in our previous studies.^7^, ^8^ Heterozygous PS2 mutant (*n* = 10, ♂5, ♀5) and PS2 wild‐type mice (*n* = 10, ♂5, ♀5) matched for age (12–18 months old) were used in accordance with the National Institute of Toxicological Research of the KFDA guidelines as well as the regulations for the care and use of laboratory animals of the animal ethics committee of Chungbuk National University (CBNUA‐1634‐21‐01). Animals were handled in an accredited KFDA animal facility in accordance with the AAALAC international animal care policies. All mice were housed in a room that was automatically maintained at 21–25°C and relative humidity (45%–65%) with a controlled light–dark cycle. Mice were sacrificed after behavioral tests by CO2 asphyxiation.

### 2.2 Morris water maze

The water maze test is a commonly accepted method for memory test, and we performed this test as described by Morris^38^ Maze testing was carried out by the SMART‐CS (Panlab) program and equipment. A circular plastic pool (height: 35 cm, diameter: 100 cm) was filled with water made opaque with skim milk kept at 22–25°C. An escape platform (height: 14.5 cm, diameter: 4.5 cm) was submerged 1–1.5 cm below the surface of the water in position. Testing trials were performed on a single platform and at two rotational starting positions. After testing trial, the mice were allowed to remain on the platform for 120 s and were then returned to their cage. Escape latency and escape distance of each mouse were monitored by a camera above the center of the pool connected to a SMART‐LD program (Panlab).

### 2.3 Probe test

To assess memory retention, a probe test was performed 24 h after the water maze test. The platform was removed from the pool which was used in the water maze test, and the mice were allowed to swim freely. The swimming pattern of each mouse was monitored and recorded for 60 s using the SMART‐LD program (Panlab). Retained spatial memory was estimated by the time spent in the target quadrant area.

### 2.4 Passive avoidance performance test

The passive avoidance test is generally accepted as a simple method for testing memory. The passive avoidance response was determined using a “step‐through” apparatus (Med Associates Inc.) that is divided into an illuminated compartment and a dark compartment (each 20.3 × 15.9 × 21.3 cm) adjoining each other through a small gate with a grid floor, 3.175 mm stainless steel rods set 8 mm apart. On the first day, the mice were placed in the illuminated compartment facing away from the dark compartment for the training trial. When the mice moved completely into the dark compartment, it received an electric shock (0.45 mA, 3 s duration). Then the mice were returned to their cage. One day after training trial, the mice were placed in the illuminated compartment and the latency period to enter the dark compartment defined as “retention” was measured. The time when the mice entered into the dark compartment was recorded and described as step‐through latency. The retention trials were set at a cutoff time limit of 3 min.

### 2.5 Open field test

The open field test (OFT) was performed according to a previously described method with minor modifications.^39^ The open field consisted of a square box with a total diameter of 60 × 60 × 45 cm, divided into 16 squares, each 15 × 15 cm. In the middle of the open field, a central zone was set up in 30 × 30 cm squares. The light (50 W) was positioned 100 cm above the floor center. Each mouse was placed in a section of the central zone and allowed to explore the environment freely for 10 min. The time spent in each zone and the total distance traveled were measured using images captured on video (SMART‐LD program; Panlab).

### 2.6 Elevated plus maze test

The elevated plus maze apparatus comprised two perpendicular open arms (30 × 5 cm) and two closed arms (30 × 5 cm) with 15‐cm‐high walls, extending from the central platform (5 × 5 cm). The maze was elevated to a height of 38 cm above floor level, as it has been validated and described.^40^ The number of entries and the time spent in each of the open and enclosed arms were recorded.

### 2.7 Novelty‐suppressed feeding

The novelty‐suppressed feeding test (NSF) is often used as a measure of depression‐like behaviors. Like the OFT, the NSF test is based on rodents' innate fear of novel spaces. However, the NSF test introduces an additional component of motivation, as the food‐deprived animal's drive to eat conflicts with its fear of novel open spaces. Mice were food‐deprived 24 h prior to the test, with free access to water and were moved to the dimly lit testing room 1–2 h before the test. Mice were placed into one corner of an open field apparatus (17 × 17 × 12 in.) with clear acrylic walls and an opaque white acrylic floor. A food pellet was placed in the center of the open field and animals were placed in one corner. Latencies to approach and to begin eating were recorded with a limit of 15 min. As soon as the mouse was observed to eat, or the 15‐min time limit was reached, the mouse was removed from the open field and placed in the home cage and observed until it began to eat in the home cage.

### 2.8 Tail suspension test

TST was performed as described in the references.^41^ Briefly, animals were suspended above the floor and recorded by a video camera for 6 min. The duration of immobile behavior was manually measured blinding to the treatment. The increase in immobility indicated depression‐like behavior.

### 2.9 Forced swim test

FST was performed as described in the references.^41^ Briefly, animals were placed in a cylinder containing water and recorded by a video camera for 6 min. The duration of climbing and immobile behaviors was manually measured in the first 2 min and last 4 min respectively blinding to the treatment. The increase in immobility and decrease in climbing both indicated depression‐like behaviors.

### 2.10 Collection and preservation of brain tissues

After behavioral tests, mice were perfused with PBS with heparin under inhaled CO2 anesthetization. The brains were immediately removed from the skulls, after that, only the hippocampus region was isolated and stored at −80°C until biochemical analysis.

### 2.11 Western blot analysis

Western blotting was performed as described. To detect target proteins, specific antibodies against BACE1 and APP (1:1000; Abcam, Inc.), DVL3, P‐GR, PSEN2 (1:500; Cell Signaling Technology, Inc.), GR, DKK‐1, t‐tau (D‐8), p‐tau (PHF‐6), p‐GSK‐3β, GSK‐3 and β‐actin (1:500; Santa Cruz Biotechnology Inc.) were used. The blots were then incubated with the corresponding conjugated secondary antibodies such as anti‐mouse, anti‐rabbit, and anti‐goat purchased from Santa Cruz Biotechnology Inc (Santa Cruz, CA, USA). Immunoreactive proteins were detected with an enhanced chemiluminescence western blotting detection system.

### 2.12 RT‐PCR

DVL3 RNA level was measured by quantitative reverse transcription polymerase chain reaction (qRT‐PCR). Total RNA was extracted using RiboEX (Geneall biotechnology, Seoul, Korea) from hippocampus tissue and cDNA was synthesized using High‐Capacity cDNA Reverse Transcription kit (Thermo Scientific). Quantitative real‐time PCR was performed on a 7500 real‐time PCR system (Applied Biosystems) for custom‐designed primers and β‐actin was used for house‐keeping control using HiPi Real‐Time PCR SYBR green master mix (ELPIS biotech). Cycling conditions consisted of initial denaturation step of 3 min at 94°C, a denaturation step of 30 s at 94°C, an annealing step of 30 s at 60°C, and extension step of a minute at 72°C followed by 40 cycles. The values obtained for the target gene expression were normalized to β‐actin and quantified relative to the expression in control samples.

Each sample was run with the following primer pairs:

β‐actin, Forward primer: 5′‐ GGCTGTATTCCCCTCCATCG‐3′, Reverse primer: 5′‐ CCAGTTGGTAACAATGCCATGT‐3′;
DVL3, Forward primer: 5′‐ GTCACCTTGGCGGACTTTAAG‐3′, Reverse primer: 5′‐ CCAAAATCGTCGTCCATAGACTT‐3′;

Along with qRT‐PCR, enzyme‐linked immune‐sorbent assay (ELISA) was used to measure DVL3 RNA level. Lysates of brain tissue were obtained through a protein extraction buffer containing protease inhibitor.

### 2.13 Immunohistochemistry

The brains were collected from mice following perfusion and immediately fixed in 4% paraformaldehyde for 24 h. The brains were transferred successively to 10%, 20%, and 30% sucrose solutions. Subsequently, brains were frozen on a cold stage and sectioned in a cryostate (20 μm‐thick). Sections were treated with endogenous peroxidase (3% H2O2 in PBS), followed by an additional two washes in PBS for 10 min each. The brain sections were blocked for 1 h in 3% bovine serum albumin (BSA) solution and incubated overnight at 4°C with Beta Amyloid 1–42 (1:500; Abcam, Inc.), tau phosphorylation at serine 396 and disheveled (DVL) segment polarity protein 3 (P‐Tau s396; 1:500; DVL3; 1:200; Cell Signaling Technology, Inc.), GSK3β phosphorylation at serine 9 (1:100; Santa Cruz Biotechnology Inc.), GR phosphorylation at serine 211, Dickkopf WNT signaling pathway inhibitor 1 (1:500; Santa Cruz Biotechnology Inc.). After incubation with the primary antibodies, brain sections were washed three times in PBS for 10 min each. After washing, brain sections were incubated for 1–2 h at room temperature with the biotinylated goat anti‐rabbit, goat anti‐mouse, or donkey anti‐goat IgG‐horseradish peroxidase (HRP) secondary antibodies (1:500; Santa Cruz Biotechnology, Inc.). Brain sections were washed three times in PBS for 10 min each and visualized by a chromogen diaminobenzidine (Vector Laboratories) reaction for up to 10 min. Finally, brain sections were dehydrated in ethanol, cleared in xylene, mounted with Permount (Fisher Scientific), and evaluated on a light microscope (Microscope Axio Imager. A2; Carl Zeiss; ×50 and ×200).

### 2.14 Measurement of tau and DVL3 levels

Lysates of brain tissue were obtained through a protein extraction buffer containing protease inhibitors. Total tau and DVL3 levels were assessed utilizing a commercially available ELISA kit obtained from (Camarillo, California, total tau: #KMB7011), (Sandiego, CA, USA, p‐tau (S396): #MBS7269992, DVL3: #MBS9363777), Protein was extracted from brain tissues using a protein extraction buffer (PRO‐PREP; Intron Biotechnology), incubated on ice for 1 h, and then centrifuged at 13,000 × *g* for 15 min at 4°C. In brief, 50 μL of the sample was added to a precoated plate and incubated for 2 h at 37°C. After removing any unbound substances, a biotin‐conjugated antibody specific for DVL3 was added to the wells. After washing, avidin‐conjugated HRP was then added to the wells. Following a wash to remove any unbound avidin‐enzyme reagent, a substrate solution was added to the wells and color developed in proportion to the amount of tau bound in the initial step. Following the addition of stop solution, the optical density was measured at 450 nm in a Molecular Devices VersaMax.

### 2.15 Serum collection and serum ELISA assay

Whole blood collected from mice was processed within 2 h of collection using the following protocol. Collected blood was centrifuged at 1000 × *g* for 15 min at room temperature, after which the serum supernatant was aspirated and transferred to a new tube. This was subsequently aliquoted into Eppendorf tubes and stored at −80°C until analyzed. An ELISA was used to measure cortisol level in the serum, according to the manufacturer's instructions.

### 2.16 Statistical analysis

All statistical analysis was performed with GraphPad Prism 4 software (version 4.03; GraphPad software, Inc.). D'Agostino & Pearson omnibus normality test and KS normality test were analyzed for all data. Unpaired two‐tailed Student's *t*‐test or two‐way ANOVA for repeated measures and Bonferroni post hoc analysis were used. All values are presented as mean ± standard error of the mean. Significance was set at *p* < 0.05 for all tests.

## 3 RESULTS

### 3.1 Alzheimer's disease‐related behaviors in PS2 knock‐in mice

**FIGURE 1:**
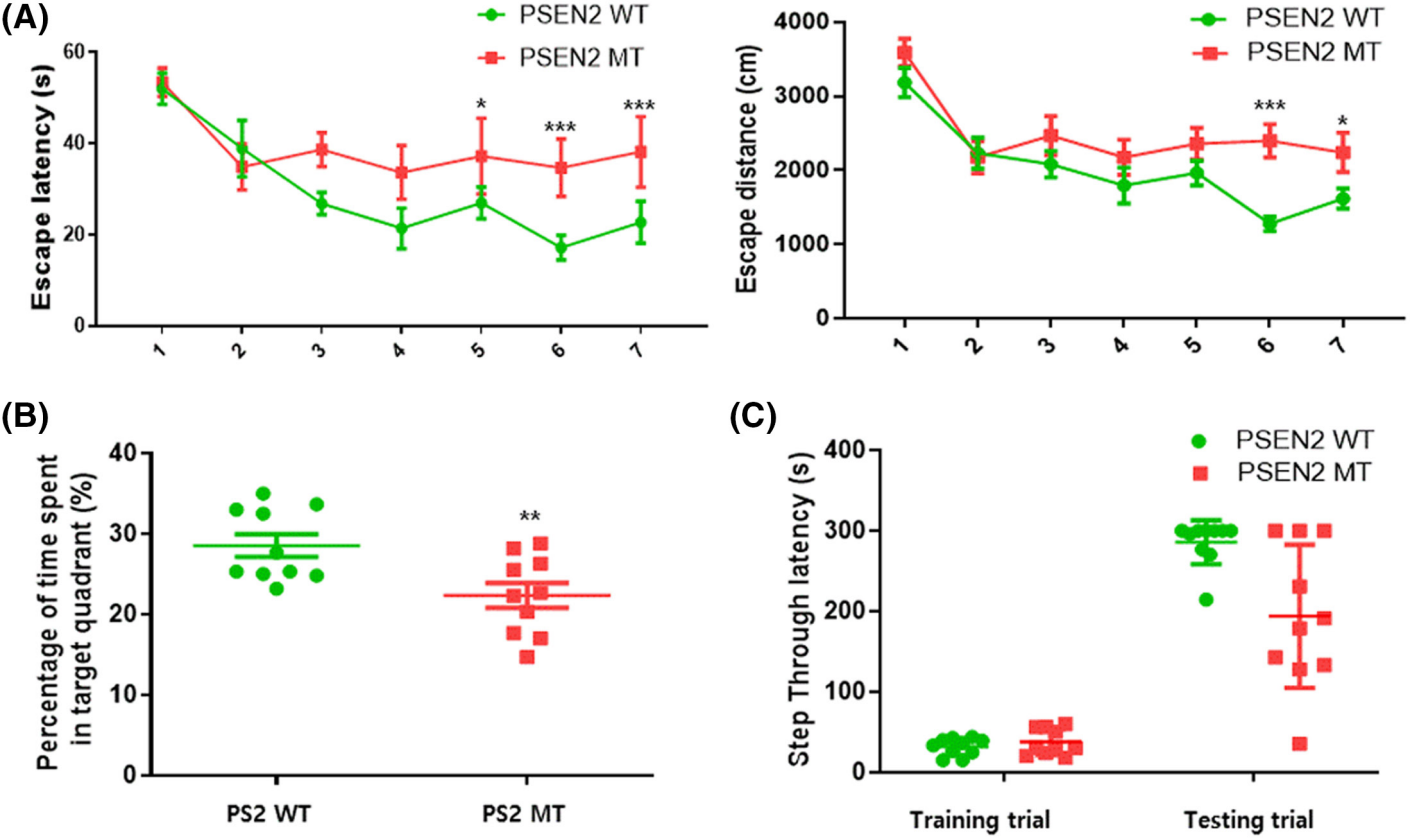
AD‐related behavior in PS2 knock‐in mice. To validate memory loss in the PS2 AD mouse model, we executed the water maze test (A), the probe test (B), and the passive avoidance test (C). Memory and learning ability in the PS2 knock‐in mouse group and the PS2 WT mouse group were evaluated using escape latencies (a, s) and escape distance (a, cm) for 7 days, and after the water maze test, measured time spent in target quadrant (b, %) by probe test for 1 day. Each value is mean ± S.E.M. from 10 mice. *, Significantly different from MT group (p < 0.05). **, Significantly different from WT group (*p* < 0.01). ***, Significantly different from WT group (*p* < 0.001).

To investigate spatial learning ability and memory loss in the PS2 AD mouse model, the Morris water maze, probe, and passive avoidance performance test were performed sequentially (Figure 1A). PS2 knock‐in mice exhibited longer escape latency and distance than PS2 WT mice. On the final day, PS2 knock‐in mice group showed an escape latency and distance of 38.11 s and 2239.44 cm, whereas the equivalent figures for the PS2 WT group mice were 22.68 s and 1619.03 cm, respectively (F(4, 33) = 28.65, *p* < 0.0001, Escape latency and F(1, 32) = 11.03, *p* = 0.0022, Escape distance, Figure 1A). The day after the water maze test (Day 7), a probe test was performed to validate the time spent in the target quadrant zone to assess memory. The time spent in the quadrant zone was increased in the PS2 WT mice (27.67%) compared to the PS2 knock‐in mice (22.35%) (*t* = 2.958, *p* = 0.0084; Figure 1B). The water maze and probe tests showed that the PS2 knock‐in mice spent more time finding the platform than the PS2 WT mice. Next, we performed a passive avoidance test to validate short‐term memory. In the training trial, no significant differences in latency were found; however, in the test trial, the PS2 WT group mice showed an increased latency (285.78 s) compared to the PS2 knock‐in group mice (193.87 s) (F(1, 18) = 7.137, *p* = 0.0156; Figure 1C). From the above results, it can be inferred that there was memory dysfunction in the PS2 knock‐in group mice.

### 3.2 Protein level of tau and Aβ in the brains of PS2 knock‐in mice

**FIGURE 2:**
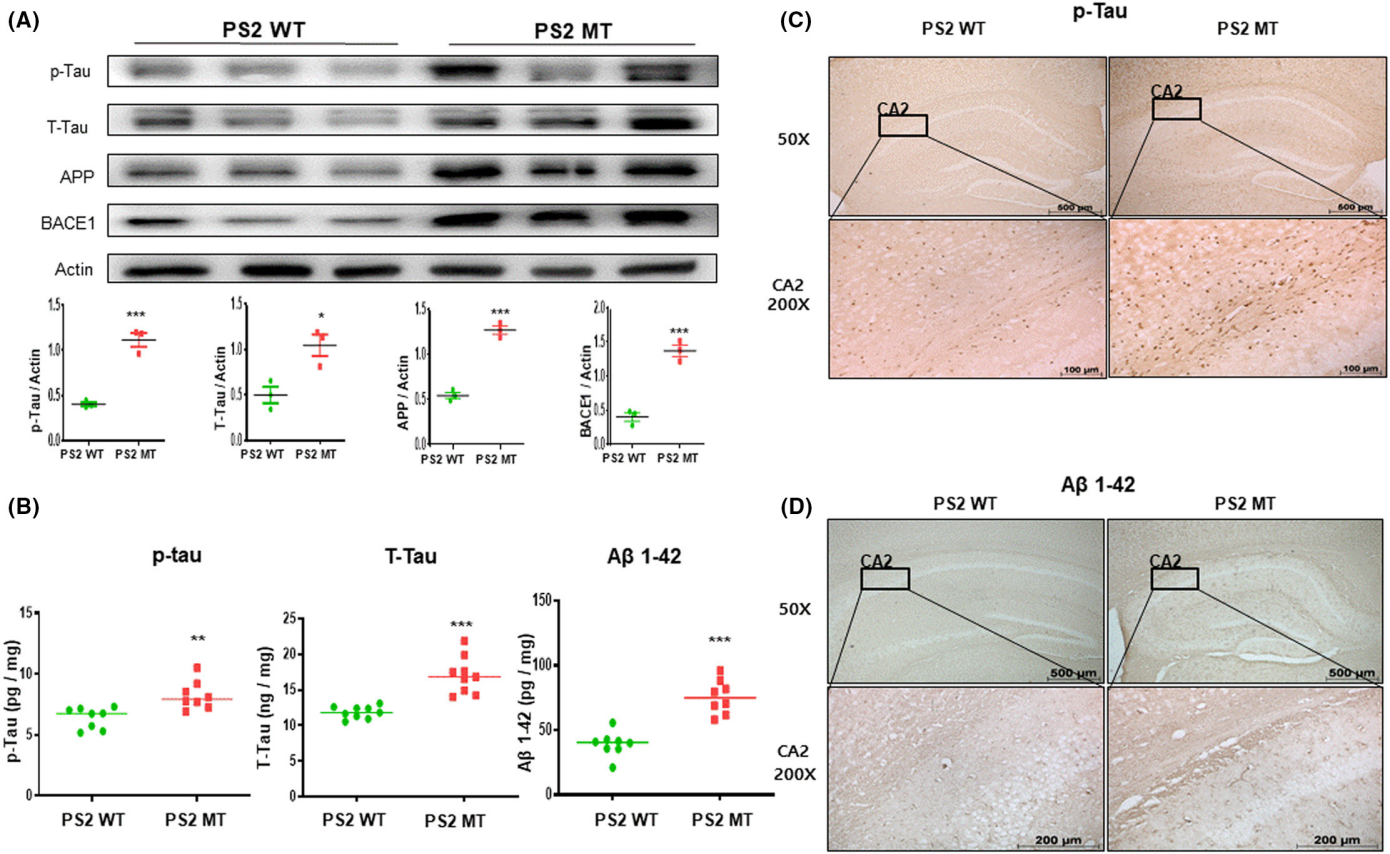
Protein level of tau and amyloid beta (Aβ) in PS2 knock‐in mice brains. To measure the levels of tau and Aβ‐related proteins, the western blot was performed on the mouse brains (A). Each of western blot value is mean ± S.E.M. from three mice. ELISA was assessed to validate protein level of total tau, phosphorylation tau, and Aβ1‐42 (B). Each value is mean ± S.E.M. from eight mice. Immunostaining of p‐tau (C) and Aβ1‐42 (D) in the hippocampus was performed in 10‐μm‐thick sections of mice brain. *, Significantly different from WT group (*p* < 0.05). **, Significantly different from WT group (*p* < 0.01). ***, Significantly different from WT group (*p* < 0.001).

The phosphorylated tau protein and Aβ are considered central mediators of AD pathogenesis. To measure AD‐related protein levels, we performed the western blot on the mouse brains. The brains of mice in the PS2 knock‐in group showed increased expression of APP (*t* = 12.60, *p* = 0.0002), BACE1 (*t* = 9.163, *p* = 0.0008), p‐tau (*t* = 8.978, *p* = 0.0009), and t‐tau (*t* = 3.687, *p* = 0.0211) compared to those of the PS2 WT mice (Figure 2A). Next, to measure the levels of total tau, p‐tau (serine 396), and Aβ, ELISA was performed in the PS2 AD mouse brains. The total tau level was up to 16.51 ng/mg in the brains of the PS2 knock‐in group mice and 11.29 ng/mg in the brains of PS2 WT mice (*t* = 3.613, *p* = 0.0028). Also, p‐tau (Serine 396) levels were up to 7.69 ng/mg in the brains of PS2 knock‐in mice and 6.73 ng/mg in the brains of PS2 WT mice (*t* = 5.697, *p* < 0.0001). Also, Aβ levels were higher in PS2 MT mice up to 16.51 and 12.4 pg/mg in the brains of PS2 WT mice (*t* = 6.355, *p* < 0.0001) (Figure 2B). To confirm the protein expression levels of phosphorylated tau and Aβ, immunohistochemistry was performed on the mouse brains. The PS2 knock‐in group mouse brains had higher expression levels of total tau and p‐tau compared to the PS2 WT group mouse brains (Figure 2C,D). Based on the above results, it is possible that memory dysfunction in PS2 knock‐in group mice was due to increased Aβ as well as tau phosphorylation (Figure 2A–D).

### 3.3 Depression‐related behavior in PS2 knock‐in mice

**FIGURE 3:**
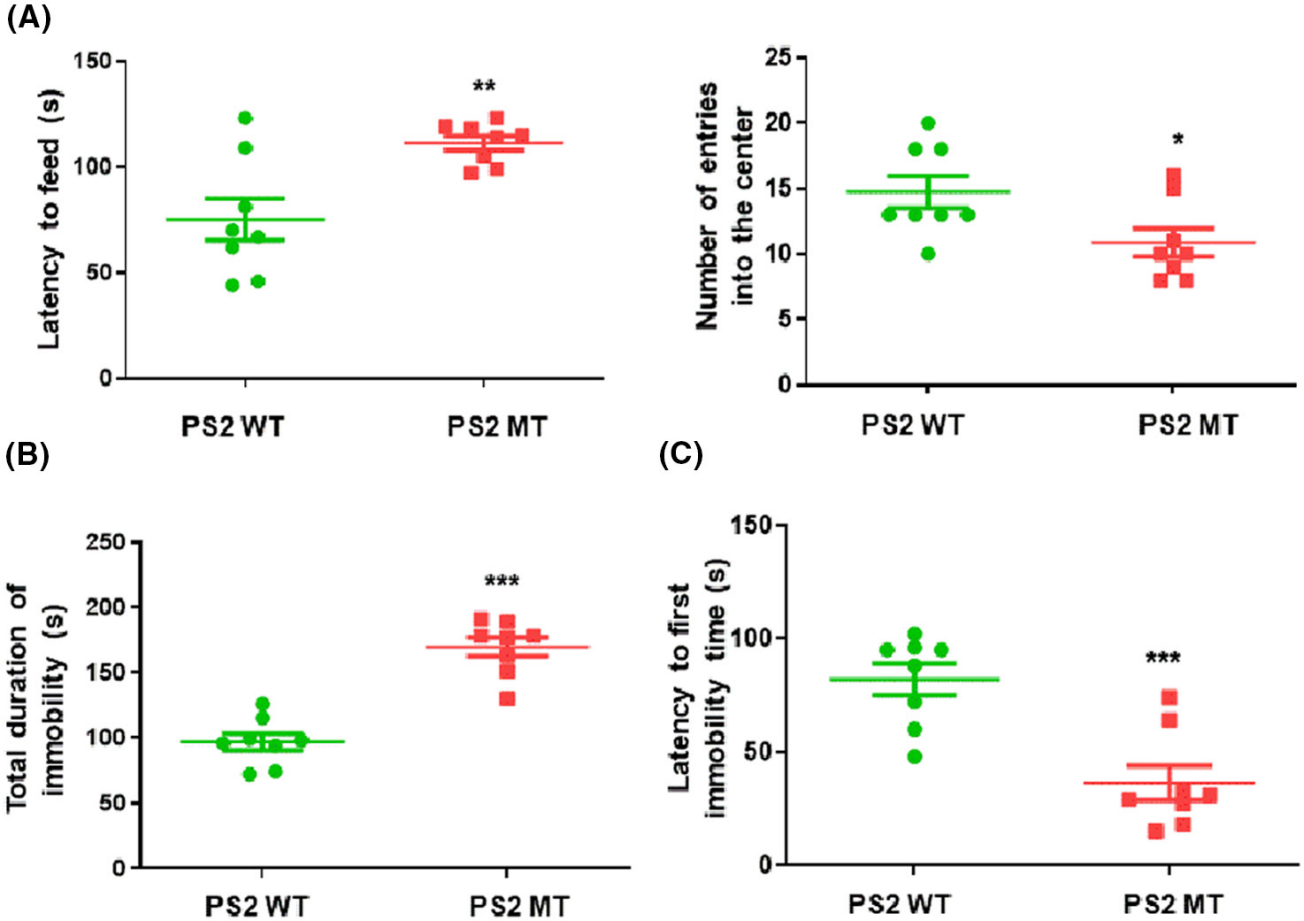
Depression‐related behavior in PS2 knock‐in mice. To validate depression‐related behavior in the PS2 AD mouse model, we executed the novelty‐suppressed feeding test (NSF) (A), tail suspension test (TST) (B), and forced swimming test (C). Depression in PS2 knock‐in mice group and PS2 WT mice group were evaluated by the total duration of latency to feed time (a, s) and number of entries into the center (a, *n*). After the NSF, the total duration of immobility in the TST was performed (c, s) Finally, the forced swimming test was performed to validate latency to first immobility time (b, s). Each value is mean ± S.E.M. from eight mice. *, Significantly different from WT group (*p* < 0.05). **, Significantly different from WT group (*p* < 0.01). ***, Significantly different from WT group (*p* < 0.001).

It is well known that chronic stress is broadly used to model anxiety as well as depression. We conducted anxiety‐related behavior in PS2 knock‐in group mice, OFT, and elevated plus maze test (EPM). The percent of the time spent in the center was higher in PS2 knock‐in group mice (2.8%) compared to PS2 WT group mice (2.5%). Also, the number of entries into the center was higher in PS2 knock‐in group mice than PS2 WT group mice (Figure S1). The day after the OFT, EPM was performed. The number of entries into the close arm was higher in PS2 WT group mice compared to PS2 knock‐in group mice, and on the contrary, the number of entries into the open arm was higher in PS2 knock‐in group mice than PS2 WT group mice (Figure S1). From the above results, anxiety behaviors were reduced in PS2 knock‐in group mice compared to PS2 WT group mice. After the anxiety‐related behavioral experiment, we conducted a depression‐related behavioral experiment. To investigate depression‐related behavior in PS2 knock‐in group mice, the NSF, tail suspension test (TST), and forced swim test (FST) were performed. Latency to take a food pellet was reduced in PS2 knock‐in group mice compared to PS2 WT group mice (*t* = 3.418, *p* = 0.0042) (Figure 3A). Also, the number of entries into the center was higher in PS2 WT group mice compared to PS2 knock‐in group mice (*t* = 2.381, *p* = 0.032) (Figure 3A). The day after the NSF, a TST was performed. The PS2 knock‐in mice were immobile for longer (163 s) than the PS2 WT mice (94 s) (*t* = 7.476, *p* < 0.0001) (Figure 3B). Next, we performed FST. The latency to the first immobility time for PS2 knock‐in mice was shorter (37.25 s) than for PS2 WT mice (80.5 s) (*t* = 4.458, *p* = 0.0005) (Figure 3C). From the above results, it was confirmed that the PS2 knock‐in mice exhibited more severe depressive behaviors.

### 3.4 Cortisol level of PS2 knock‐in mice brains and serum

**FIGURE 4:**
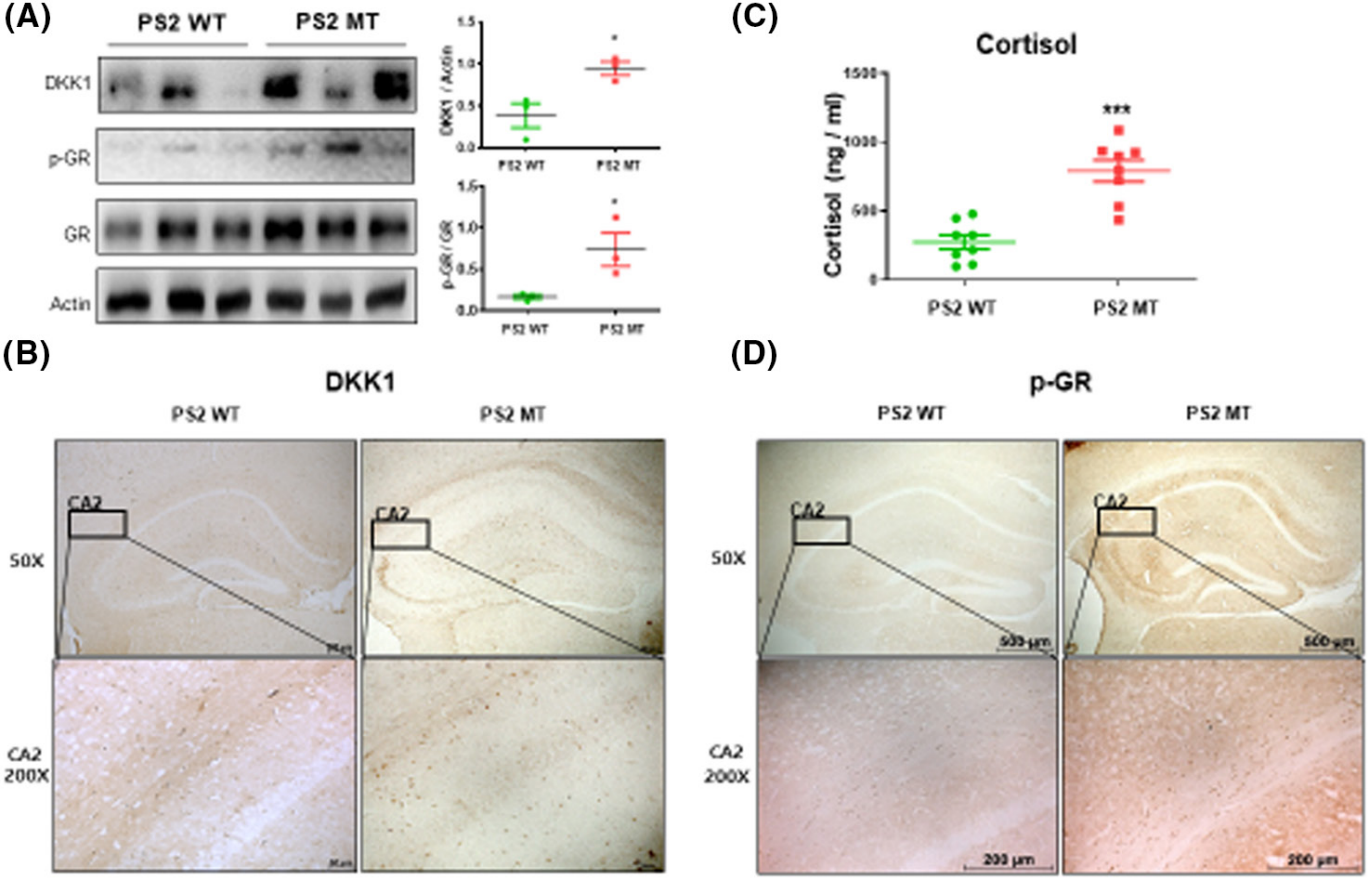
Cortisol level of PS2 knock‐in mice brains and serum. Western blotting was performed to validate depression‐related protein expression levels (A). Each of western blot value is mean ± S.E.M. from three mice. Immunostaining of Dickkopf‐1 (B) and p‐GR (D) in the hippocampus was performed in 10‐μm‐thick sections of mice brain. ELISA was assessed to validate cortisol level on mice serum (C). Each value of cortisol level is mean ± S.E.M. from eight mice. *, Significantly different from WT group (*p* < 0.05). **, Significantly different from WT group (*p* < 0.01). ***, Significantly different from WT group (*p* < 0.001).

Stress is associated with the onset of depressive episodes and cortisol hypersecretion is considered a biological risk factor of depression.^11^ Moreover, glucocorticoids induce DKK‐1 in the central nervous system, which suggests that DKK‐1 induction and the ensuing inhibition of the canonical Wnt pathway is a critical component of the molecular cascade leading to hippocampal damage in response to stress.^11^ To measure cortisol‐related proteins, p‐GR, GR, and DKK‐1 levels, we performed the western blot on the mouse brains. The PS2 knock‐in mice had increased levels of p‐GR (*t* = 2.809, *p* = 0.0484) and DKK‐1 (*t* = 3.394, *p* = 0.0274) compared to the PS2 WT mice (Figure 4A). To confirm the protein expression levels of phosphorylated GR and DKK‐1, immunohistochemistry was performed on the mouse brains. The PS2 knock‐in mice showed higher expression levels than the PS2 WT mice (Figure 4B,D). Next, we measured the cortisol level in the serum of PS2 knock‐in mice. The cortisol level was approximately 792.77 ng/mL in the serum of the PS2 knock‐in group mice, but in the serum of the PS2 WT mice, it was 218.55 ng/mL (*t* = 5.587, *p* < 0.0001) (Figure 4C). Therefore, our results indicated that depressive behavior in the PS2 knock‐in mice was associated with higher corticoid levels.

### 3.5 Expression of DVL3 was decreased in the brains of PS2 knock‐in mice

**FIGURE 5:**
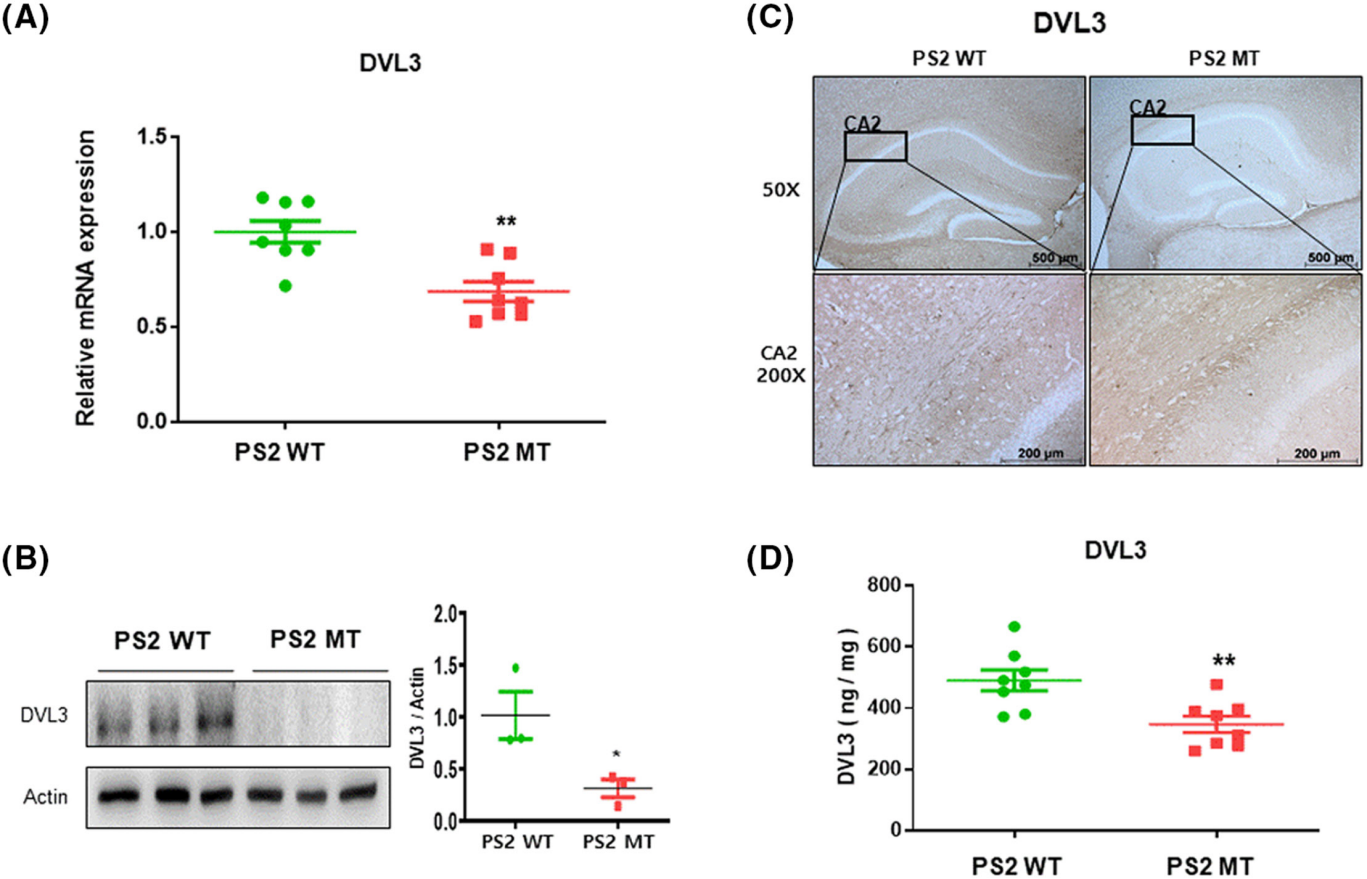
Expression of disheveled (DVL3) was decreased in PS2 knock‐in mice brains. The mRNA expression level of DVL3 was measured by RT‐PCR (A). To validate the DVL3 level, western blotting was performed (B). Western blot value is mean ± S.E.M. from three mice. Immunostaining of DVL3 in the hippocampus was performed in 10‐μm‐thick sections of mice brain (C). ELISA was assessed to validate protein levels of DVL3 (D). Each value is mean ± S.E.M. from eight samples. *, Significantly different from WT group (*p* < 0.05). **, Significantly different from WT group (*p* < 0.01).

The role of the DVL3 signaling pathway in mood disorders is well known.^42^, ^43^, ^44^ Genome‐wide association studies (GWAS) have shown that DVL3 polymorphism interacts with MDD susceptibility.^45^ Other research has shown that DVL3 regulates the metabolism of APP.^46^ Moreover, overexpression of the DVL protein inhibits GSK3β‐mediated phosphorylation of tau in transfected CHO cells.^47^ To measure the expression level of DVL3, we measured the mRNA expression level of DVL3. RT‐PCR was performed on the brains of the PS2 knock‐in and PS2 WT mice. The mRNA level of DVL3 was lower in the PS2 knock‐in group than in the PS2 WT group (*t* = 4.053, *p* = 0.0012) (Figure 5A). To confirm the protein expression levels of DVL3, we performed western blot on the mouse brains. The PS2 knock‐in mouse brains showed reduced levels of protein expression of DVL3 compared to the PS2 WT mouse brains (*t* = 2.889, *p* = 0.0446) (Figure 5B). We also performed immunohistochemistry on the mouse brains. The brains of the PS2 knock‐in group showed lower levels of expression of DVL3 than those of the PS2 WT group (*t* = 3.311, *p* = 0.0051) (Figure 5C). Next, ELISA was performed to confirm the expression levels of DVL3. The PS2 knock‐in group mice brains showed decreased protein expression levels compared to the PS2 WT group mice brains (Figure 5D). Therefore, our results indicate that decreased levels of DVL3 could provide a link between AD and depression.

### 3.6 DVL3 expression was related to GSK3β protein expression

**FIGURE 6:**
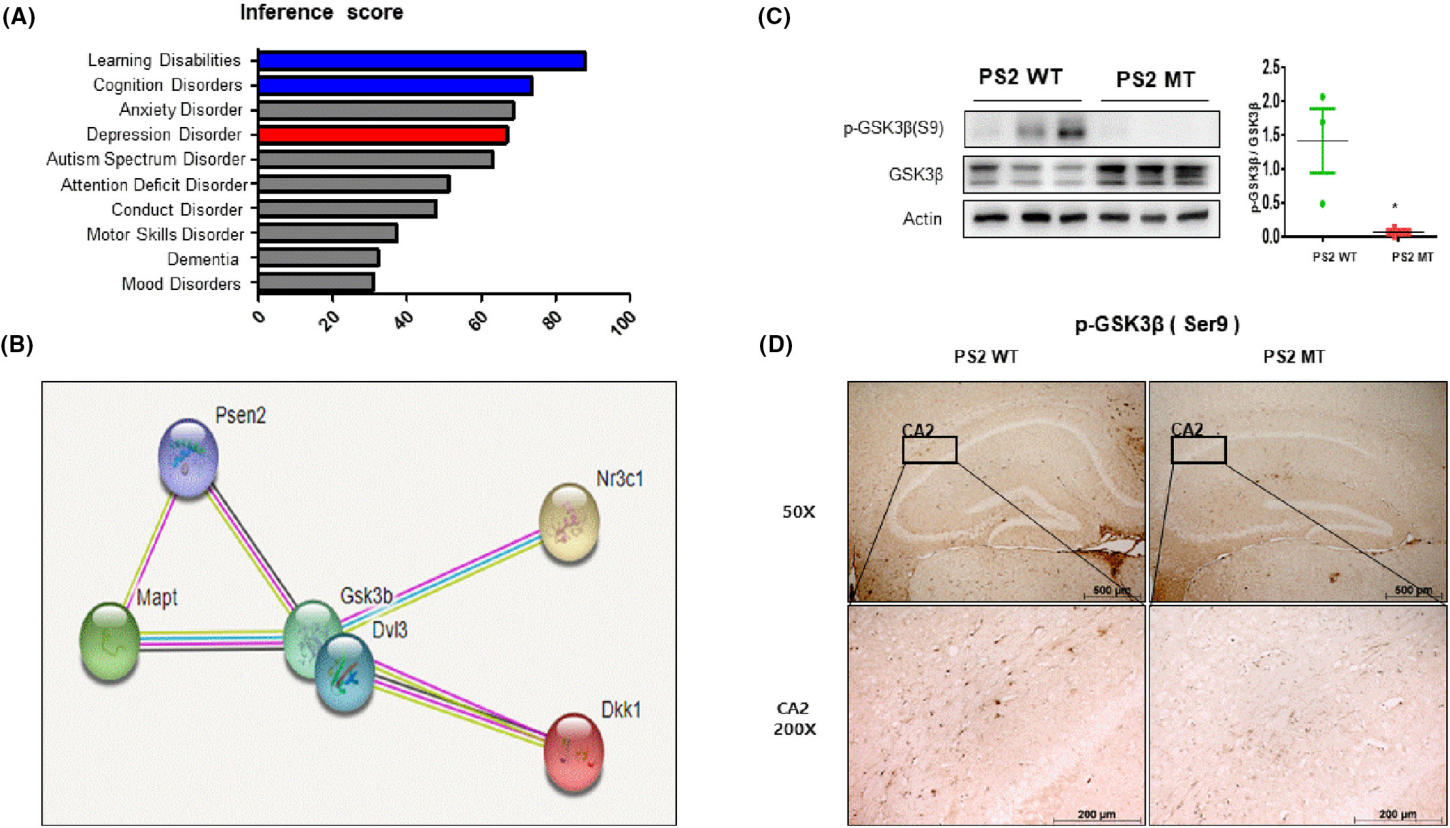
Disheveled (DVL3) expression was related to GSK3β protein expression. The CTD database was used to determine the relationship between DVL3, AD, and depression (A). The STRING analysis database was used to find gene–gene interactions between DVL3, AD‐related proteins, and depression‐related proteins (B). Western blotting was performed to verify protein levels of p‐GSK3β (Ser9) (C). Immunostaining of p‐GSK3β (Ser9) in the hippocampus was performed in 10‐μm‐thick sections (D). Each value is mean ± S.E.M. from three mice. *, Significantly different from WT group (*p* < 0.05). **, Significantly different from WT group (*p* < 0.01). ***, Significantly different from WT group (*p* < 0.001).

Previous studies have shown that GSK3β is associated with AD and depression. It is also well known that DVL inhibits the activation of GSK3β. In the next part of our study, we focused on the relationship between DVL3 and GSK3β phosphorylation. To figure out the relationship between DVL3, AD, and depression, we used the comparetive toxicogenomics database (CTD) (http://ctdbase.org). The inference scores of DVL3 related to learning disabilities and cognition disorders were 88.03 and 73.34, respectively, and depressive disorder scored 66.78 (Figure 6A). We also searched for gene–gene relationships between DVL3 and AD‐related proteins such as APP and microtubule‐associated protein tau (MAPT), and also depression‐related proteins such as NR3C1 and DKK‐1, by using STRING (https://string‐db.org). We predicted the STRING analysis would show that DVL3 is closely related to GSK3β. GSK3β is closely associated with the AD proteins PS2 and MAPT and with the depression‐related proteins NR3C1 and DKK‐1 (Figure 6B). Based on the STRING analysis, we determined the level of GSK3β expression and its phosphorylation since it is very closely related to DVL3 and downregulated proteins. There were higher levels of both GSK3β and GSK3β phosphorylation in the brains of the PS2 knock‐in mice than in the brains of the PS2 WT mice (*t* = 2.818, *p* = 0.0479) (Figure 6C). Immunohistochemistry analysis also showed a higher level of GSK3β expression in the brains of PS2 knock‐in mice (Figure 6D). Therefore, our results indicate that a reduction in DVL3 expression downregulates GSK3β, which prevents AD and depression in PS2 MT mice.

## 4 DISCUSSION

The purpose of our study was to find a link between AD and depression, and also to find a possible pathway connecting the two diseases. From our AD‐related behavioral test, PS2 knock‐in mice appear to have much more severe memory dysfunction than PS2 WT mice. Moreover, there was increased expression of tau and Aβ proteins in the brains of PS2 knock‐in mice. We also examined depression‐related behavioral tests in PS2 knock‐in mice. Our results indicate that PS2 knock‐in mice are more prone to depressive behaviors than PS2 WT mice. We measured cortisol levels and cortisol‐related protein levels in PS2 knock‐in mouse brains and found them to be higher than those in PS2 WT mouse brains, which indicates that depression is more severe in PS2 AD knock‐in mice. These results indicate that depression development could be associated with AD development.

We further investigated the possible pathway connecting the two diseases by using the CTD and the STRING database. Using the CTD database, we found that DVL3 was closely related to both AD and depression. Other research shows that the interaction of DVL with the Wnt signal could be related to neurological diseases including AD and depression^48^ Other research shows that DVL3 levels were high when rats were exposed to chronic forced swim stress.^34^ Moreover, stress raises cortisol levels and makes depression more severe.^49^ It was also demonstrated that the mRNA and protein levels of DVL3 in healthy individuals are significantly different from those of depressed individuals.^50^ In addition, DVL3 is involved in the pathogenesis of MDD in female Chinese Han patients.^32^ In agreement with these data, we also found that the cortisol levels of PS2 knock‐in mice were higher than those of PS2 WT mice. These results show that DVL3 can act as an important common target protein for AD and depression development.

Accumulation of Aβ and tau phosphorylation are the most representative hypotheses for the underlying disease mechanism in AD.^51^ Both hypotheses are associated with GSK3β. Activation of GSK3β mediates Aβ‐induced neuritic damage in AD.^52^ Moreover, GSK3 inhibitors reduced Aβ pathology and ameliorated cognitive decline in an AD mouse model.^53^ Other research evidence shows that GSK3β increases tau phosphorylation.^54^, ^55^ Moreover, increased activity of GSK3β could accumulate hyperphosphorylation of tau molecules abnormally in AD.^23^ Our in vivo studies showed that there was a lower level of phosphorylation of GSK3β and DVL3 in PS2 knock‐in mice than in PS2 WT mice. On the contrary, the levels of total tau, phosphorylation of tau, and APP were elevated in PS2 knock‐in mice. Moreover, both depression and AD have connections with GSK3β and DVL3. Increased GSK3β activity has been found in the platelets of depressed patients.^19^ Other research has demonstrated the downregulation of phosphorylated GSK3β in the nucleus accumbens in the mouse social defeat model of depression.^37^

Our research on both AD‐related behavioral tests and depression‐related behavioral tests shows that memory dysfunction and depression were more severe in PS2 knock‐in mice than in PS2 WT mice. Also, we confirmed that levels of tau, Aβ, and cortisol were higher in the brains of PS2 knock‐in mice. Moreover, we know that DVL3 is associated with two diseases: AD and depression. As the expression of DVL3 was decreased in PS2 knock‐in mice, the expression of AD and depression‐related proteins were increased. Moreover, DVL3 suppresses the expression of major proteins associated with AD and depression by inhibiting the activity of GSK3β. These data suggest a possible mechanism for the comorbidity of depression and AD, and indicate that AD and depression could be associated, and DVL3 may serve as a critical target for the development of AD and depression.

## AUTHOR CONTRIBUTIONS

**Seung Sik Yoo:** Conceptualization, data curation, formal analysis, validation, roles/writing—original draft, writing—review and editing. **Dong Won Lee:** Conceptualization, validation. **Hyeon Joo Ham:** Conceptualization, writing—review and editing. **In Jun Yeo:** Conceptualization, writing—review and editing. **Ju Young Chang:** Conceptualization, validation. **Jaesuk Yun:** Conceptualization, validation, writing—review and editing. **Dong Ju Son:** Formal analysis, conceptualization. **Sang‐Bae Han:** Writing—review and editing. **Jin Tae Hong:** Resources; conceptualization; supervision; funding acquisition; writing—original draft; writing—review and editing. All authors contributed to writing—review and editing.

## CONFLICT OF INTEREST STATEMENT

The authors have no conflict of interest to report.

## CONSENT

Consent for publication was obtained from all authors.

## Supporting information


Figure S1.

## Data Availability

The data that support the findings of this study are available from the corresponding author upon reasonable request. Some data may not be made available because of privacy or ethical restrictions.
