# Supplementary figures and images for: Presenilin‐2 knock‐In mice show severe depressive behavior via DVL3 downregulation
Source: CNS Neurosci Ther. 2023 Jul 27;30(2):e14370. doi: 10.1111/cns.14370 (PMC10848049; doi:10.1111/cns.14370)

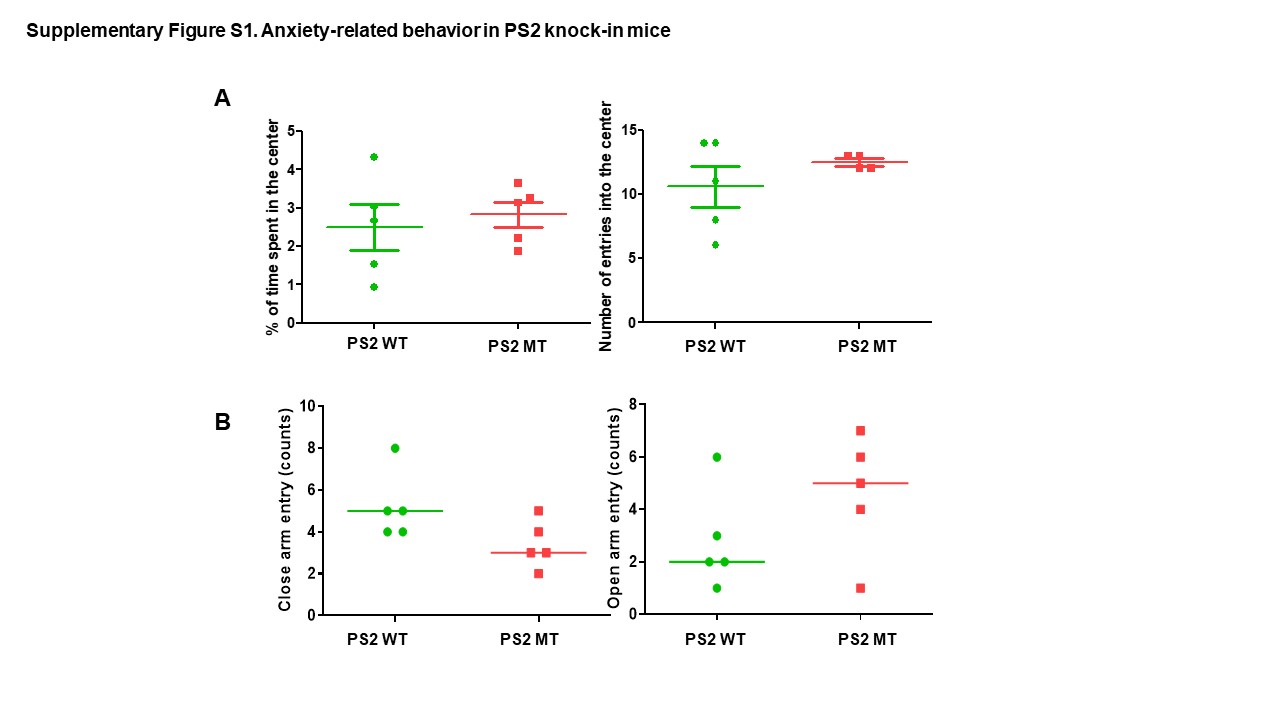

Supplement: Supplementary file 1 — Figure S1. [file CNS-30-e14370-s001.jpeg]
